# Detecting Animal Contacts—A Deep Learning-Based Pig Detection and Tracking Approach for the Quantification of Social Contacts

**DOI:** 10.3390/s21227512

**Published:** 2021-11-12

**Authors:** Martin Wutke, Felix Heinrich, Pronaya Prosun Das, Anita Lange, Maria Gentz, Imke Traulsen, Friederike K. Warns, Armin Otto Schmitt, Mehmet Gültas

**Affiliations:** 1 Breeding Informatics Group, Department of Animal Sciences, Georg-August University, Margarethe von Wrangell-Weg 7, 37075 Göttingen, Germany; felix.heinrich@uni-goettingen.de (F.H.); armin.schmitt@uni-goettingen.de (A.O.S.); 2 Livestock Systems, Department of Animal Sciences, Georg-August University, Albrecht-Thaer-Weg 3, 37075 Göttingen, Germany; anita.lange@agr.uni-goettingen.de (A.L.); maria.gentz@thuenen.de (M.G.); imke.traulsen@uni-goettingen.de (I.T.); 3 Bioinformatics Group, Fraunhofer Institute for Toxicology and Experimental Medicine (Fraunhofer ITEM), Nikolai-Fuchs-Str. 1, 30625 Hannover, Germany; pronaya.prosun.das@item.fraunhofer.de; 4 Agricultural Test and Education Centre House Düsse, Chamber of Agriculture North Rhine-Westphalia, Haus Düsse 2, 59505 Bad Sassendorf, Germany; Friederike.Warns@LWK.NRW.DE; 5 Center for Integrated Breeding Research (CiBreed), Georg-August University, Albrecht-Thaer-Weg 3, 37075 Göttingen, Germany; 6 Statistics and Data Science, Faculty of Agriculture, South Westphalia University of Applied Sciences, 59494 Soest, Germany

**Keywords:** pig detection, pig tracking, convolutional neural network, Kalman filter, precision livestock farming

## Abstract

The identification of social interactions is of fundamental importance for animal behavioral studies, addressing numerous problems like investigating the influence of social hierarchical structures or the drivers of agonistic behavioral disorders. However, the majority of previous studies often rely on manual determination of the number and types of social encounters by direct observation which requires a large amount of personnel and economical efforts. To overcome this limitation and increase research efficiency and, thus, contribute to animal welfare in the long term, we propose in this study a framework for the automated identification of social contacts. In this framework, we apply a convolutional neural network (CNN) to detect the location and orientation of pigs within a video and track their movement trajectories over a period of time using a Kalman filter (KF) algorithm. Based on the tracking information, we automatically identify social contacts in the form of head–head and head–tail contacts. Moreover, by using the individual animal IDs, we construct a network of social contacts as the final output. We evaluated the performance of our framework based on two distinct test sets for pig detection and tracking. Consequently, we achieved a Sensitivity, Precision, and F1-score of 94.2%, 95.4%, and 95.1%, respectively, and a MOTA score of 94.4%. The findings of this study demonstrate the effectiveness of our keypoint-based tracking-by-detection strategy and can be applied to enhance animal monitoring systems.

## 1. Introduction

Today, it is well known that domestic pigs are highly social animals, maintaining hierarchical structures and socially organized groups. In commercial farming systems, the established social orders are frequently disrupted due to mixing groups as they are transferred between different housing and production stages [1,2]. Mixing of unaquainted animals leads to the establishment of a new social hierarchy going along with agonistic interactions which may result in reduced animal welfare and health [3,4,5].

In order to enhance animal welfare and health in future husbandry systems, the analysis of animal interactions as well as their monitoring and prediction is of high importance in research and commercial farming. Reasons for agonistic or aggressive behavior are manifold [2,6,7,8,9,10], contribute to a certain extent to the animal specific behavior, and also include a high variation between animals [2].

Nowadays, video recordings are a standard tool in research for observing pig pens due to their non-invasive nature. Recent technological advances including deep learning techniques, led to the rise of precision livestock farming applications to partially automate the time consuming video evaluation process [11]. Within the area of precision livestock farming, the tasks of multiple object detection and motion tracking have been studied intensively in recent years, in order to remotely monitor several animals and to capture the animals activity [11,12,13,14,15]. While multiple object detection refers to the task of locating several objects belonging to a category of interest within an image [16], multiple object tracking can be described as tracing the movement of objects throughout a consecutive number of video frames and consistently assigning individual object IDs [17].

With the recent advances in the area of deep learning, convolutional neural network (CNN)-based applications achieved state-of-the-art results in various image and video object detection scenarios [18]. Here, the most frequently used detection approaches aim to localize an object of interest by computing a bounding box around the object [19,20,21]. Although these approaches work successfully for various problem settings, due to the overlapping of the predicted bounding boxes, their applicability is limited for the analysis of videos with high utilization rates and several pigs in a close environment [22,23]. Furthermore, the standard bounding box approach only provides the positional information without taking the orientation of the animal into account which is a key information in order to reliably differentiate distinctive contact types like head–head interactions.

To overcome this limitation, an alternative detection approach was developed by Psota et al. [23]. The authors proposed a keypoint-based CNN for the detection of individual body parts of pigs. After processing the CNN output with a cross-check matching algorithm for assigning the individual body parts, they were able to successfully differentiate multiple animals even in a close proximity environment. Achieving a sensitivity, precision rate and an F1-score of 96%, 100%, and 98%, respectively, their approach proved to be highly successful in identifying the location and orientation of individual animals. As an extension, Psota et al. [11] applied this method to deal with the problem of tracking individual animals by using a second object detection CNN to detect ear tags which serve as a pig-individual identifier. Although this approach shows a lot of practical potential, using individual ear tags requires additional effort for the attachment of the ear-tags. Furthermore, the detectability of the corresponding ear-tags must be ensured, as the visibility of the tags is often prevented by heavy interactions between the animals, bad lighting conditions or a high degree of pollution in the pig compartment. As a possible solution, it seems beneficial to use the detected body parts directly for animal tracking which could increase the applicability of the keypoint-based detection approach, while simultaneously reducing the complexity without the need to train a second CNN for object detection.

Therefore, by following the idea in [23] we implement a CNN based framework for detecting individual body parts of pigs and use the predicted shoulder-tail information directly as the input for a Kalman filter (KF)-based tracking algorithm. The KF is currently one of the most frequently used approaches for tracking the motion activity of multiple objects within a video [24,25] which allows the assignment of individual animal IDs in this study. Subsequently, by collecting the shoulder-tail information as well as the animal ID in our framework we differentiate between specific head–head and head–tail contacts. As a result of this, our framework is able to determine a table of social contacts and to compute a graphical network of the social relationships. Such type of contacts could provide crucial information about social interactions including tail and ear biting. Consequently, using the proposed framework we aim to automate the process of video data analysis by quantifying the number of social encounters for several pigs within a video sequence. This information can then be used by researchers to specifically analyze scenes of interest within their respective fields or to directly perform a SNA.

The remainder of this article is structured as follows. Section 2 describes the data used for this analysis and explains the methodical foundation as well as the evaluation rationale applied in this article. Next, the results for the animal detection, animal tracking, and social contact identification are presented and discussed in Section 3. Section 4 concludes this article.

## 2. Materials and Methods

**Figure 1:**
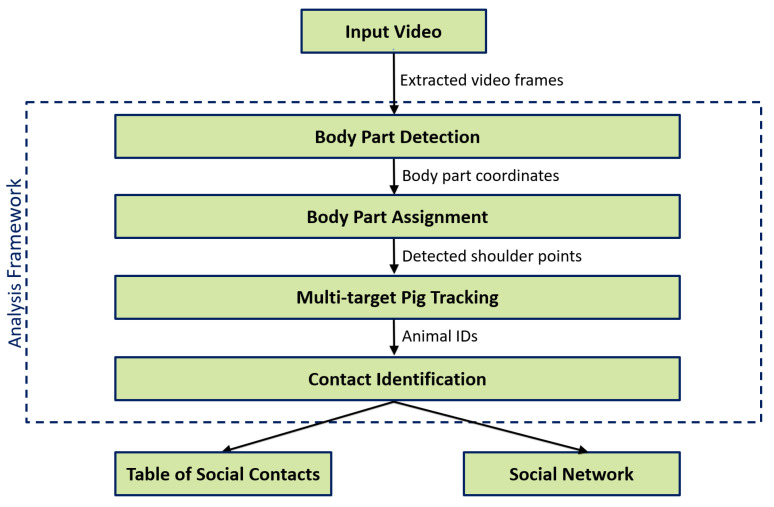
Flowchart of the analysis applied in this study.

In this section, the data used for the analysis as well as the different stages of the proposed method and the evaluation rationale are described in detail. The underlying multi-stage framework of the proposed method is illustrated in Figure 1.

The proposed method follows a tracking-by-detection (TBD) approach with the goal of tracking a known number of pigs within the pig compartment. As the input signal, a video sequence represented by a series of consecutive frames $S=(s_{1},s_{2},\ldots ,s_{N})$ is used, where *N* is the number of frames. In this context, TBD refers to first detecting objects of interest in each video frame using a pre-trained detector and then linking the independent detections at the temporal dimension over a longer period of frames [26,27].

In this study, the location and orientation of each pig within the video is determined frame-wise using a keypoint-based CNN to output the coordinates of important body parts (shoulder, tail, left ear, and right ear). After associating the body parts and assigning a unique ID to each pig, the shoulder coordinates in time *T* are used as the input signal for a Kalman filter to predict and track the location of future shoulder positions in T+i with i=(1,2,…,N−T). By tracking the shoulder points, two distinct types of body part contacts are identified as being either a head-head or head-tail contact. If two shoulder points are close to each other, the encounter is marked as a head-head contact. If a shoulder point is close to a tail point, the encounter is marked as a head–tail contact. Finally, by incorporating the frame number information, animal IDs and types of contact, a table of social contacts as well as a graphical representation in form of a social network is constructed as an output.

### 2.1. Data Acquisition and Processing

**Figure 2:**
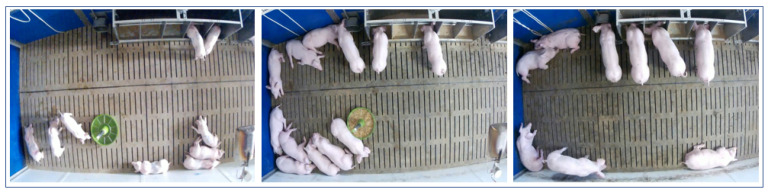
Example frames of the pig compartment under investigation with a known number of pigs.

The video data used for this study was collected by [28,29] between October and December 2018 at the research farm Futterkamp of the Chamber of Agriculture of Schleswig-Holstein in Germany during a research project to investigate the effects of different farrowing and rearing systems on the stress level of piglets. For this purpose, a single static camera of the type AXIS M3024-LVE (Axis Communications AB, Lund, Sweden) was assembled 3 m above the ground which recorded all videos with a frame-per-second (fps) rate of 10 frames and a display resolution of 1280 × 800 pixels. For this study, sequences with a varying number of animals and a fixed camera angle have been selected. Figure 2 shows three example frames of structurally identical pens.

In our analysis, we extracted all video frames and converted them to a grayscale format with a pixel dimension of 640 × 400 pixels, in order to avoid a potential bias of the CNN by differentiating between day and night recordings [30]. The dimensionality reduction was carried out to reduce CNN training time and, thereby, increase the computational efficiency.

### 2.2. Pig Detection

**Figure 3:**
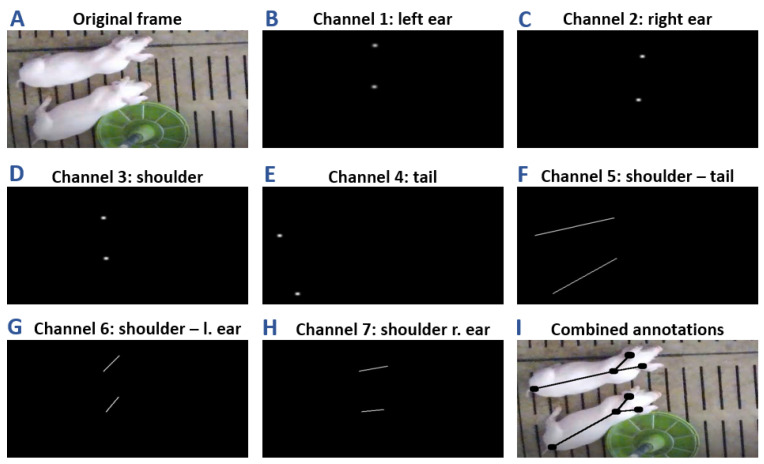
(**A**) The original image which serves as the input for the CNN. (**B**–**H**) The corresponding ground truth annotations containing the positional body part information which are used for the training process. (**I**) The original image combined with the ground truth annotations.

An essential step in the tracking of individual pigs is their successful detection. For this purpose, Psota et al. [23] established a keypoint-based CNN to detect distinct body parts of pigs and highlighted the advantage of this approach over existing bounding box detections. Using this keypoint approach, we implemented a CNN to receive a video frame and to output the coordinates of four individual body parts for each animal. Similar to the work in [23], we stored the coordinate information of the left ear, right ear, shoulder and tail point directly as a binary image in a separate image channel (Figure 3B–E). Additionally, the information for the connection lines shoulder–tail, shoulder–left ear, and the shoulder–right ear are included (Figure 3F–H). In comparison to conventional top-down detection methods, which output bounding box or ellipsoid coordinates, the detected keypoints directly provide a pose representation which facilitates the contact identification of the animals [23].

**Figure 4:**
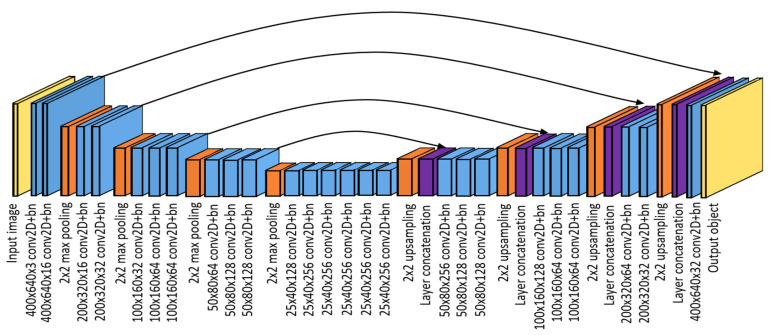
The implemented CNN follows an autoencoder structure to create the seven-channel output object given a gray-scaled video frame. For each convolutional layer the dimensional information is given in the format height × width × number of convolutional filters.

During the training process, a CNN is trained to map the input image to the ground truth annotations by highlighting the important pixels of the corresponding body parts. The architecture of our CNN follows an autoencoder structure which is illustrated in Figure 4.

The CNN consists of 25 convolutional layers combined with 2×2 max pooling and upsampling layers. The first ten layers are used to reduce the input dimension from 640 × 400 pixels to a latent representation of 40 × 25 pixels and extract the main features for the body part detection. At the lowest dimension, six stacked convolutional layers forward the latent image representation to a set of upsampling and convolutional layers, which step-wise increase the image dimension back to 640 × 400 pixels and output the approximate body part coordinates. After each upsampling layer, a residual connection with a concatenation layer is used to copy a representation from the encoder layers to the decoder layers to decrease the reconstruction loss and improve the training efficiency [23,31]. All convolutional layers are implemented with a ReLU activation function, zero padding and a stride parameter of 1. Using the Adam optimizer [32] and binary cross-entropy loss, the network applies a sigmoid activation for the last layer to output pixel intensities between 0 and 1.

The CNN was implemented in Python (version 3.7.6) [33] using the deep learning framework Keras (version 2.2.4) [34] with TensorFlow (version 2.0) [35] as a backend. The model training was carried out on a workstation equipped with two Intel Xeon Gold 6138 CPUs, 512 GB RAM, and a NVIDIA Quadro P5000 GPU.

Subsequently, to train the CNN, we annotated a training data set consisting of 2457 images. To increase the overall sample size and to enable the model to see more heterogenous animal postures, we augmented the training images as well as the corresponding ground truth annotations using vertical and horizontal shifting, shrinking and image rotation. After augmentation, the total training data set had a size of 12,285 images of which 90% were used for training and 10% for the model validation after each epoch.

**Figure 5:**
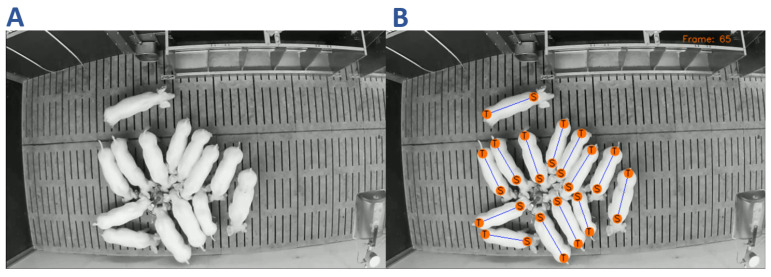
Example of the CNN pig detection showing the original frame (**A**) and the detected shoulder and tail points (**B**). The frame shows a feeding situation in which several pigs are in close proximity to each other. Each pig is marked by highlighting the corresponding shoulder and tail points as well as the connection line.

After the CNN has learned to predict the positions of the individual body parts in the two-dimensional image space, the location and orientation of each pig are determined based on the CNN output. For this step, we mainly focus on the analysis of Channel 5 (Figure 3F), as it mainly carried the most accurate and robust information. By extracting the start and end coordinates of each shoulder-tail line, a depth-first-search algorithm (DFS) [36] is applied to determine the pigs location. However, Channel 5 does not contain the information of the animal’s orientation. Therefore, we incorporate the information of Channels 1–4 to identify shoulder and tail points of each pig. An example frame after the detection process is given in Figure 5.

### 2.3. Pig Tracking

After determining the location and orientation of each pig, this step aims to track the pigs’ movement and to link their trajectories over the total sequence of frames. While previous approaches mainly focused on pig tracking using bounding box detections [19,37,38,39], the suitability of these approaches is limited to identify social contacts in close proximities because they do not incorporate the animals’ orientation and show a high risk of ID switches in situations of overlapping boxes. To reduce this limitation, we apply for the first time a combination of a KF tracking approach [40] with the body part detections as the input signal to track individual animals and to determine distinct contact types. While the CNN output can still contain false detections, we use the KF as an unsupervised, dynamic model to estimate and track the shoulder positions, even in frames in which the true point could not be detected by the CNN.

The KF process is divided into two phases: a prediction and an update phase. In the prediction phase, a prediction of the shoulder position for the current time step *k* is computed based on the KF estimate of the shoulder point of the previous time step k−1. During the update phase, new CNN detections at *k* are used to adjust the current prediction and to compute the KF estimate at time *k*, which is used as new input for the prediction phase of the next time step k+1. In the case of false positive or false negative CNN detections, the input signal variance is increased, which leads the KF to weight down the importance of the CNN input and to increase the weight of the previous KF prediction [41]. Consequently, the KF yields a more robust estimate of the shoulder point coordinates which overcomes the problem of shoulder–tail swaps and misdetections.

**Figure 6:**
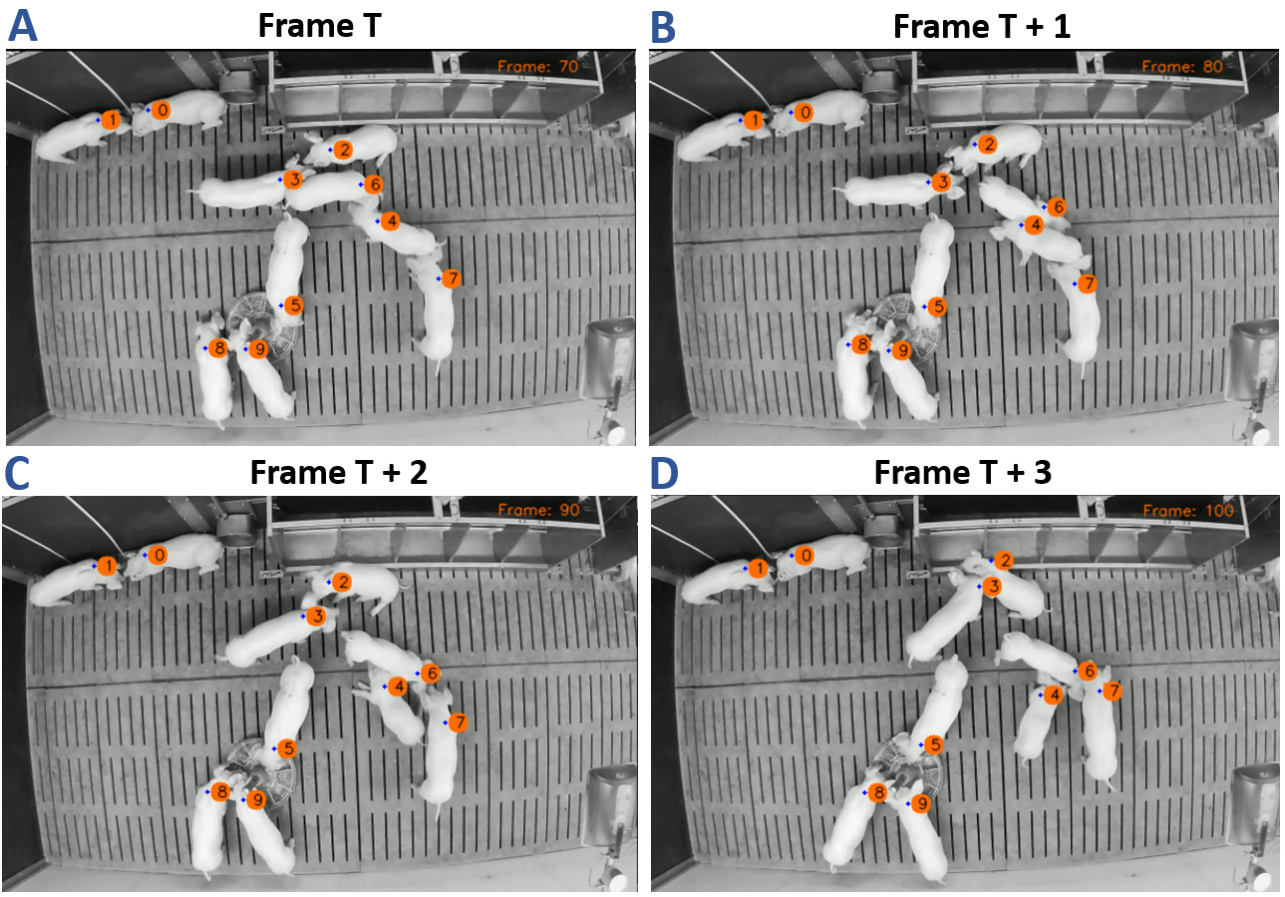
The multi-target pig tracking, shown for an example sequence of four frames, on a one second interval. The frame number is printed on the top, and the fps-rate was set to ten frames. The KF estimate of the shoulder point is highlighted by the blue dot, near the shoulder region of each pig. The corresponding ID is placed right of it.

After the KF is initialized, it is applied to the ordered shoulder points produced by the CNN. While all videos have been recorded with a fps rate of 10 frames, even intense motion changes of the animals caused just slight pixel variations in the consecutive frames. Therefore, a KF shoulder point in frame *k* is mapped to the corresponding KF shoulder point in frame k+1 by minimizing the Euclidean distance. Consequently, each KF shoulder estimate is assigned an individual animal ID. An example of the pig tracking and ID assignment is shown in Figure 6.

### 2.4. Identifying Contact Information

**Figure 7:**
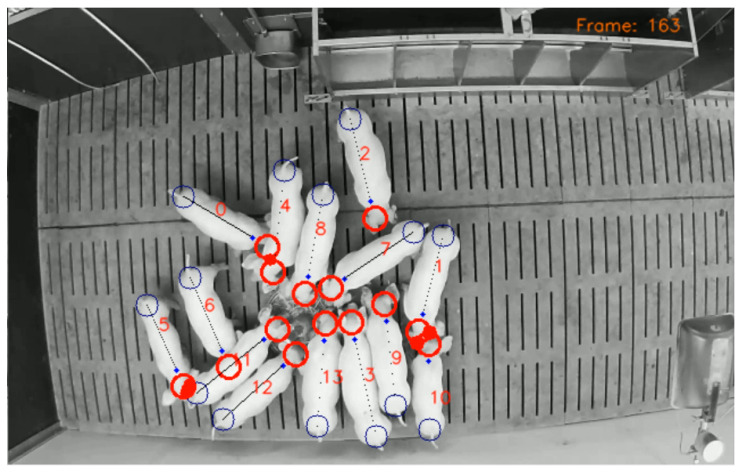
Based on the KF estimates, a region of interest for each head and tail area is computed. By detecting the intersection of at least two regions, the type of contact and the associated animals can be identified. Exemplarily, a head–tail contact can be detected between pigs 5 and 11 and a head–head contact for pigs 1 and 10.

The last stage of our proposed framework aims to identify animal contacts in the form of either head–head or head–tail contacts, which may be related to tail biting or nosing behavior. We use the KF shoulder and tail estimates, as well as the pig orientation to define a region of interest at the head and tail area of each pig. If at least two pigs are nearby to each other so that either both head regions or one head and one tail region are sufficiently close, the head and tail regions are intersecting, which indicates a potential contact. To account for different age and size levels of the animals, the average length of each shoulder–tail line per frame is calculated and used to scale the head and tail regions to a radius *r*, defined as:

(1) $$r=\frac{1}{\alpha N}\sum_{i=1}^{N}\sqrt{{\left(s_{i}-t_{i}\right)}^{2}}$$

where $s_{i}$ and $t_{i}$ are the shoulder and tail coordinates of the i-th pig, *N* the total number of pigs in the given frame, and α a scaling factor. In this study, an α value of 3 was empirically deemed to be optimal for computing an area of interest large enough to cover the essential part of the head and tail region, but being small enough to avoid potential false detections in the form of animals walking by. Figure 7 shows an example of the region computation and contact identification.

### 2.5. Pig Detection and Tracking Evaluation Rationale

In order to assess the overall performance of our framework, we evaluated both the CNN detection stage as well as the multi-target pig tracking stage separately. For the CNN detection, we additionally annotated 100 randomly selected images and used these frames as a test set to evaluate the CNN’s ability to predict the location of individual pigs by detecting their shoulder points. To avoid confusion we used the subscript “*D*” and “*T*” to differentiate between the detection evaluation and the tracking evaluation. For the detection stage, we computed the number of *True Positives* ($TP_{D}$), *False Positives* ($FP_{D}$), and *False Negatives* ($FN_{D}$) over all test images and calculated the *Sensitivity*, *Precision* rate, and *F1-score* defined as

(2) $$Sensitivity=\frac{TP_{D}}{P_{D}}$$

(3) $$Precision=\frac{TP_{D}}{TP_{D}+FP_{D}}$$

(4) $$F1=\frac{2TP_{D}}{2TP_{D}+FP_{D}+FN_{D}}$$

In order to determine $TP_{D}$, $FP_{D}$, and $FN_{D}$, a circular detection region around the true shoulder point was defined by computing the average distance from the true shoulder point to the true left and right ear points over all pigs of the given frame as the radius. If exactly one shoulder point was predicted by the CNN within the detection region, this point has been classified as $TP_{D}$. If more than one point was predicted within the region or outside the detection region, these points have been classified as $FP_{D}$. If no point was detected within the region, the point was classified as $FN_{D}$.

Despite the recent advances of multiple object tracking applications, there is still a lack of large-scale benchmarks and comparable evaluation metrics [42,43]. While the majority of existing object tracking publications applies a bounding box approach using the intersection over union (IoU) as an evaluation criterion between the annotated and predicted box, our proposed framework applies a keypoint-based approach, for which the IoU is not suitable. Therefore, we followed previous studies [21,38,44] and calculated the *Multiple Object Tracking Accuracy* (*MOTA*) defined as

(5) $$MOTA=1-\frac{FP_{T}+FN_{T}+IDSW}{N_{CNN}}$$

by manually determining the number of falsely tracked pigs ($FP_{T}$), pigs which have not been tracked ($FN_{T}$), the number of ID switches (IDSW), and the number of pigs detected by the CNN ($N_{CNN}$). While the total tracking error can be further divided into detection errors, association errors, and localization errors [42,45], $FP_{T}$ and $FN_{T}$ account for the detection errors and IDSW accounts for the association errors. If a detected pig is not tracked by the KF tracker, it is classified as $FN_{T}$. If the tracker tracks something different than a pig, it is classified as an $FP_{T}$. We further increased the number of $FP_{T}$ to account for the localization errors if a pig is tracked, but the corresponding tracking point is too far away from the target point. To determine the MOTA value we randomly selected 70 videos as test sequences with an average length of 20 seconds. These sequences have been analyzed by the CNN in advance, in order to obtain the coordinates of the detected body parts.

## 3. Results and Discussion

By applying our pig detection and tracking framework, we first analyzed in this study several video frames including different scenarios, in order to assess the detection and tracking performance. After that, three distinct animal contact network visualizations are presented and discussed to demonstrate the applicability and functionality of our framework.

### 3.1. Pig Detection and Tracking

As the performance of a tracking-by-detection (TBD) algorithm depends strongly on the accuracy of the corresponding detector [46], we evaluated the CNN performance for locating the pig position and determining its orientation using the *Sensitivity*, *Precision*, and *F1-Score* metric, respectively. For this purpose, we analyzed the manually annotated detection test set containing 100 randomly selected frames. Consequently, in total 1054 shoulder points have been annotated and considered for the evaluation analysis (see Table 1). In a following step, we focused on the tracking ability of the implemented KF and used 70 randomly selected video sequences as the tracking evaluation data. The results for the detection as well as for the tracking are provided in Table 1. The data sets used for the detection and tracking evaluation are made publicly available at https://github.com/MartinWut/Supp_DetAnIn (accessed on 9 November 2021).

**Figure 8:**
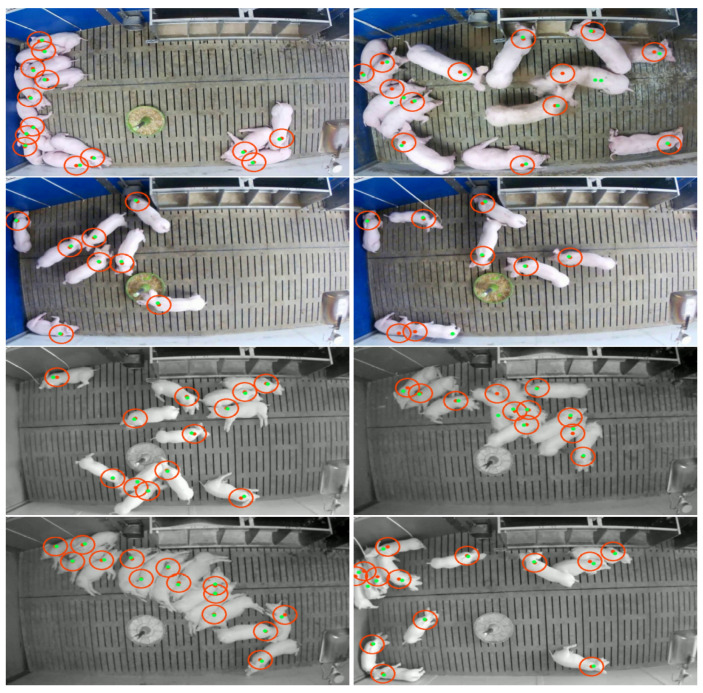
Example frames from the detection test set for day and night frames showing cases of a completely successful pig detection (**left column**) and cases in which at least one pig was not detected correctly (**right column**). A true shoulder point is highlighted by a red dot in the middle of the detection region (red circle). A green dot represents the predicted shoulder point from the CNN.

Table 1 shows that the majority of shoulder points was successfully detected by the CNN resulting in high performance values, in terms of *Sensitivity*, *Precision*, and *F1-Score*. While in total 1054 shoulder points have been manually annotated, only a relatively small fraction of these points have not been detected. On the other hand, the number of falsely detected pigs (FP =51) indicates that the detection CNN still has limitations in challenging situations like object occlusion. Figure 8 illustrates several example frames from the test set showing cases of successful and failed detections.

In Figure 8, it can be observed that the CNN successfully detected most of the pigs. Focusing on the failed detections, the large majority of failures was produced in situations in which one pig has been occluded by another pig, which led to a false negative detection. However, the problem of object occlusion and the resulting degradation in performance is not linked to the design of this study. In fact, the issue of object detection under the influence of occlusion is a challenging task which negatively affects the robustness of most detection algorithms [47]. While current approaches aim to tackle this problem by applying a compositional neural network structure in combination with an occluder model [47,48,49,50], the majority of approaches focus on the problem of partial occlusion and would, therefore, be of limited suitability for this study. Moreover, during the tracking process, the negative effect of object occlusion can be reduced to some extent, by applying a predictive model like the KF algorithm, which internally interprets the CNN detections as noisy measurement information. In cases of extreme volatility, like the loss of an object due to occlusion, the KF reduces the influence of the measurement input by increasing the importance of the Kalman prediction [51,52]. To reduce the effect of a missing shoulder or tail point to some extent, we computed the number of detected objects frame-wise and marked the corresponding frames as corrupted in cases of missing detections. Corrupted frames have then been excluded for the KF tracking where we used the previous KF estimates as the new measurement input instead.

**Figure 9:**
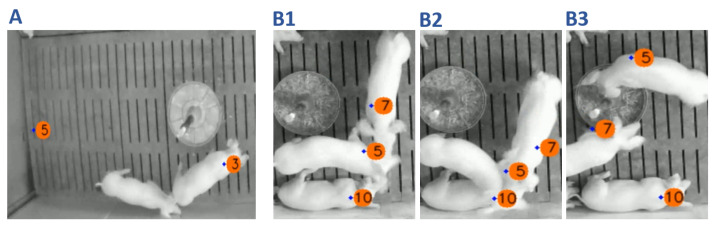
Examples of a failed tracking performance. (**A**) While the shoulder point of the lower pig was not tracked (FN), the corresponding ID was computed at an empty spot in the compartment (FP). (**B1**–**B3**) A case of an ID switch due to a close proximity interaction and the occlusion of an animal. Before the interaction takes place, ID 5 and ID 7 have been assigned correctly (**B1**). After the interaction the IDs switched so that the ID 5 was assigned ID 7 and vice versa (**B3**).

To assess the tracking performance, we further analyzed 70 test sequences containing various scenarios like feeding, resting, or interactions. As it can be seen in Table 1, the implemented KF was able to track 640 out of 678 shoulder points correctly resulting in a MOTA score of 94.4%. However, in 38 cases the tracking of the detected shoulder points failed: (i) 20 animals have not been tracked, (ii) eight animals have been tracked at the wrong position, and (iii) ten cases occurred in which the assigned ID of two pigs switched. Examples of a FP-track, a FN-track, and an ID switch are given in Figure 9.

In line with previous studies [26], we observed that if the detector is able to detect all pigs correctly, the corresponding pig tracking is working without producing corrupted tracks. In contrast, if the detection of the body parts fails, the tracker predicts the shoulder point based on the movement pattern of the previous frames. While the consequences are minor for short periods of detection failures, longer phases of missing detections lead to the effect of misplaced tracking IDs. However, this limitation is not specific to this study. Although the most successful tracking approaches are based on a TBD-strategy, the consequence of missing detections can be a significant reduction in their performance [53]. An example of a misplaced tracking ID is given in Figure 9A.

Another fundamental issue in the field of multiple object tracking, is the problem of ID switches, which is shown in Figure 9(B1–B3) [21,39,44,54]. In Figure 9(B1), the pigs with IDs 5, 7, and 10 are successfully detected and tracked. During the sequence, pigs 5 and 7 are occluding the shoulder point of pig 10, which leads to a missed detection of this animal by the detector (Figure 9(B2)). However, after all shoulder points reappear in the video, the KF tracker estimates the position of the shoulder points, but swaps IDs 5 and 7 (Figure 9(B3)).

Unlike previous bounding box-based studies, the problem of ID switches in our study only occurs in cases when the shoulder points of the animals disappear due to different obstacles, thus preventing the detection of these points. While several existing bounding box tracking applications suffer from ID switches arising from highly overlapping boxes [39], the keypoint-based approach applied in this study considers a much smaller tracking area. Therefore, a strong overlap of two or more tracked shoulder points is less likely to occur, which explains the relatively low number of ID switches given in Table 1. Of particular interest, we further studied all cases of ID switches in the test set in order to establish the main reason for the ID switch issue. We found that nine out of ten ID switches have been caused by a missed detection rather than by two detected IDs in a short distance. Only in one case a false detection caused the ID switch.

### 3.2. Animal-to-Animal Contact Identification

**Figure 10:**
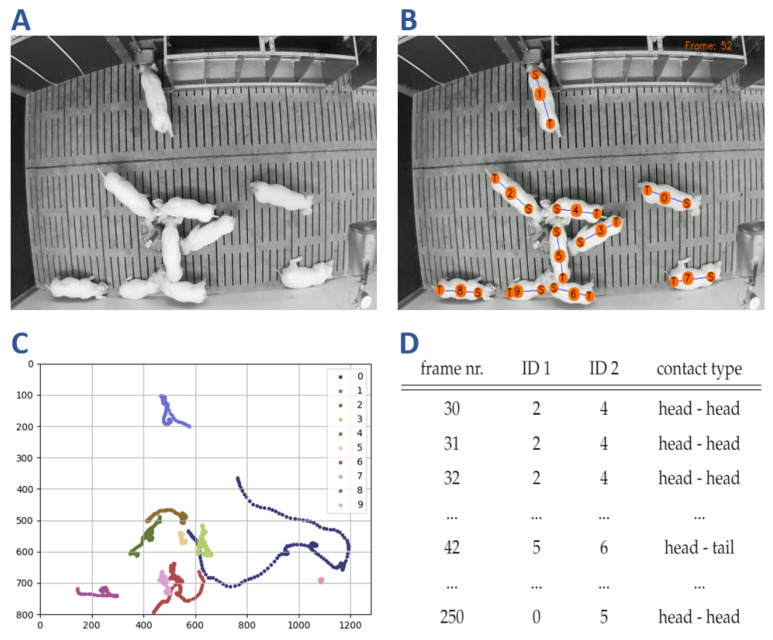
The social contact identification starts with a raw video frame (**A**). After detecting the shoulder and tail position (**B**), the trajectories are computed and analyzed over time (**C**). By identifying cases of close distances, a table of social contacts is constructed automatically (**D**).

The knowledge about social interactions is fundamental to enhance farming conditions and animal welfare. Thus, the final stage of the framework proposed in this study aims to identify such behavior patterns based on pig interactions. In particular, by focusing on close proximity contacts between at least two animals, our framework automatically takes into account head–head as well as head–tail contacts. After that, based on these contact information, we construct a trajectory map to highlight individual movement patterns, which finally provides an information table about the social contacts. An example of the identification process for one test sequence is given in Figure 10.

**Figure 11:**
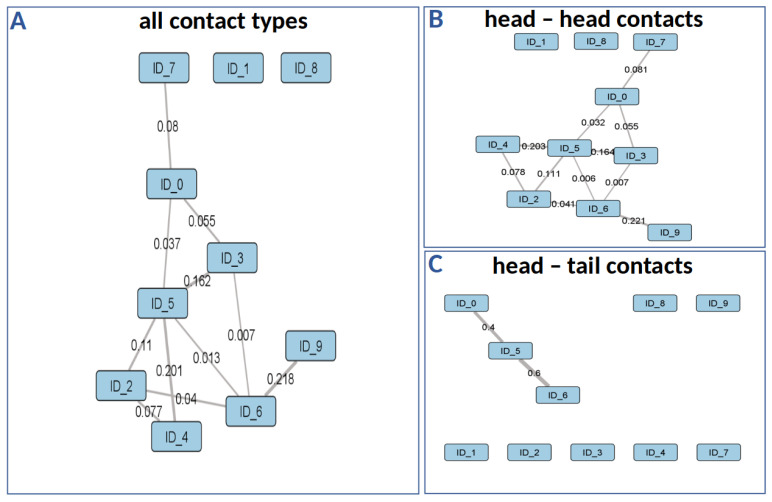
Examples of three social networks from one test sequence.

The table of social contacts (Figure 10D) contains highly essential information about the contact pattern of animals over all video frames. As Smith et al. [55] pointed out, these data are crucial in the field of behavioral ecology and the automatic contact identification can reduce observer bias as a limiting factor. To address different research questions like the investigation of hierarchical structures, agonistic behavior patterns or pen utilization, the necessary information can be derived from this table as required. Further, this table can be used for extracting a distinct contact period by restricting the frame and ID information. However, the aim of this study is to differentiate between individual contacts. Therefore, the extraction process primarily focuses on the contact type information which is used to visualize the social relationships of the observed animals. Figure 11 shows an example for the visualization depicting both type of contacts (Figure 11A) as well as the specific head–head and head–tail contact types (Figure 11B,C).

Each of the three social networks in Figure 11 is constructed by using the individual animal ID as the node information and the contact frequency as an intensity score for the edge weight. In particular, for a holistic analysis, the consideration of all contact types is crucial to establish the general contact patterns between all animals in a compartment (Figure 11A). On the other hand, with regard to specific research questions [56,57,58], a more differentiated network design focusing on a distinct type of contact can be advantageous (Figure 11B,C). As a result, the structure of the specific network types differs from the holistic network which arises from the alterations in the edge weights.

In comparison to previous studies which aim to tackle the issue of identifying social interactions by following a bounding box approach [59], our strategy is not restricted to specific behavioral patterns like escaping and chasing motion activities. Even in challenging situations like resting behavior where most of the pigs are lying down in a very small area, our proposed framework is able to identify social contacts to a certain extent.

To further extend the performance of the proposed framework, future work could focus on the implementation of more sophisticated network topologies. In this regard, recent studies [60,61] successfully showed the potential of attention mechanisms, introduced by Bahdanau et al. [62], to leverage the power of deep learning for highlighting important features. Ghaffarian et al. [60] performed a literature review analyzing 176 articles focusing on image classification, object detection, and change detection. As a result, the authors concluded that the majority of deep learning-based research studies reported a performance increase when applying an attention mechanism. This improvement could have the potential to further reduce the number of false positive predicted body parts and could enable additional filtering steps to detect corrupted video frames.

## 4. Conclusions

Today, the usage of video technology for animal monitoring is well established. However, extracting useful information is often challenging and thus limiting the potential of animal video analysis. In this study, we propose a framework for the automatic detection of social contacts to address the limitations of animal behavioral studies. By applying a keypoint-based body part detection and a subsequent pig tracking algorithm, we are able to determine the time, the animals involved, and the type of a social contact. We further process the information to construct a social network based on the contact type. To the best of our knowledge, this is the first study incorporating both a body part detection CNN as well as a Kalman filter tracking algorithm to identify social contacts. Our findings show the applicability of our approach to monitor a known number of pigs which can be used as part of early warning systems for the detection of behavioral changes. Overall, we suggest that our framework is applicable for different livestock animal monitoring systems.

## Figures and Tables

**Table 1:** Evaluation results for the pig detection set and tracking set.

Test Set	TP	FP	FN	IDSW	Sensitivity (%)	Precision (%)	F1 (%)	MOTA (%)
Detection	1019	51	35	-	94.2	95.4	95.1	-
Tracking	640	20	8	10	-	-	-	94.4

## Data Availability

The data sets used for the detection and tracking evaluation are made publicly available at https://github.com/MartinWut/Supp_DetAnIn (accessed on 9 November 2021).

## Abbreviations

The following abbreviations are used in this manuscript:

CNN	Convolutional neural network
KF	Kalman filter
SNA	Social network analysis
TBD	Tracking-by-detection
